# Supplementary material for: Heparin-based hydrogel scaffolding alters the transcriptomic profile and increases the chemoresistance of MDA-MB-231 triple-negative breast cancer cells
Source: Biomater Sci. 2020 Feb 13;8(10):2786–96. doi: 10.1039/c9bm01481k (PMC7497406; doi:10.1039/c9bm01481k)
Supplement: Supplementary file 2 [file BM-008-C9BM01481K-s002.zip › Supplementary File 4/EGFvControl/Pathways/my_analysis.Gsea.1545200981068/gsea_report_for_EGF_1545200981068.html]

Report for EGF 1545200981068 [GSEA]

| GS  follow link to MSigDB | GS DETAILS | SIZE | ES | NES | NOM p-val | FDR q-val | FWER p-val | RANK AT MAX | LEADING EDGE || 1 | HALLMARK\_E2F\_TARGETS | Details ... | 192 | 0.68 | 3.15 | 0.000 | 0.000 | 0.000 | 4041 | tags=77%, list=21%, signal=97% |
| 2 | HALLMARK\_G2M\_CHECKPOINT | Details ... | 190 | 0.62 | 2.89 | 0.000 | 0.000 | 0.000 | 5055 | tags=73%, list=26%, signal=98% |
| 3 | HALLMARK\_MYC\_TARGETS\_V1 | Details ... | 189 | 0.63 | 2.89 | 0.000 | 0.000 | 0.000 | 4803 | tags=74%, list=25%, signal=97% |
| 4 | HALLMARK\_MYC\_TARGETS\_V2 | Details ... | 56 | 0.73 | 2.78 | 0.000 | 0.000 | 0.000 | 2578 | tags=70%, list=13%, signal=80% |
| 5 | HALLMARK\_SPERMATOGENESIS | Details ... | 76 | 0.45 | 1.80 | 0.000 | 0.001 | 0.013 | 3780 | tags=42%, list=20%, signal=52% |
| 6 | HALLMARK\_MTORC1\_SIGNALING | Details ... | 190 | 0.37 | 1.70 | 0.000 | 0.004 | 0.039 | 2856 | tags=34%, list=15%, signal=39% |
| 7 | HALLMARK\_UNFOLDED\_PROTEIN\_RESPONSE | Details ... | 105 | 0.39 | 1.65 | 0.003 | 0.006 | 0.068 | 5433 | tags=44%, list=28%, signal=61% |
| 8 | HALLMARK\_DNA\_REPAIR | Details ... | 139 | 0.37 | 1.64 | 0.002 | 0.005 | 0.073 | 3726 | tags=36%, list=19%, signal=44% |
| 9 | HALLMARK\_OXIDATIVE\_PHOSPHORYLATION | Details ... | 194 | 0.35 | 1.62 | 0.000 | 0.007 | 0.101 | 5606 | tags=44%, list=29%, signal=61% |
| 10 | HALLMARK\_MITOTIC\_SPINDLE | Details ... | 189 | 0.31 | 1.44 | 0.008 | 0.037 | 0.474 | 5421 | tags=42%, list=28%, signal=58% |
| 11 | HALLMARK\_PI3K\_AKT\_MTOR\_SIGNALING | Details ... | 89 | 0.31 | 1.25 | 0.127 | 0.157 | 0.962 | 4914 | tags=35%, list=26%, signal=47% |
| 12 | HALLMARK\_UV\_RESPONSE\_UP | Details ... | 131 | 0.28 | 1.23 | 0.100 | 0.173 | 0.982 | 5265 | tags=40%, list=27%, signal=54% |
| 13 | HALLMARK\_REACTIVE\_OXIGEN\_SPECIES\_PATHWAY | Details ... | 44 | 0.31 | 1.11 | 0.282 | 0.344 | 0.999 | 3652 | tags=32%, list=19%, signal=39% |
| 14 | HALLMARK\_FATTY\_ACID\_METABOLISM | Details ... | 131 | 0.25 | 1.08 | 0.304 | 0.386 | 1.000 | 5226 | tags=31%, list=27%, signal=43% |
| 15 | HALLMARK\_UV\_RESPONSE\_DN | Details ... | 129 | 0.22 | 0.97 | 0.534 | 0.633 | 1.000 | 4787 | tags=35%, list=25%, signal=46% |
| 16 | HALLMARK\_ADIPOGENESIS | Details ... | 176 | 0.19 | 0.85 | 0.822 | 0.881 | 1.000 | 4883 | tags=28%, list=25%, signal=37% |
| 17 | HALLMARK\_PROTEIN\_SECRETION | Details ... | 94 | 0.18 | 0.77 | 0.923 | 0.976 | 1.000 | 5522 | tags=31%, list=29%, signal=43% |
| 18 | HALLMARK\_PANCREAS\_BETA\_CELLS | Details ... | 18 | 0.25 | 0.75 | 0.819 | 0.939 | 1.000 | 4618 | tags=33%, list=24%, signal=44% |
Table: Gene sets enriched in phenotype **EGF (2 samples) **[plain text format]****
